# Increased adiponectin levels are associated with higher radiographic scores in the knee joint, but not in the hand joint

**DOI:** 10.1038/s41598-021-81513-z

**Published:** 2021-01-19

**Authors:** Haimuzi Xu, Ji-Hyoun Kang, Sung-Eun Choi, Dong-Jin Park, Sun-Seog Kweon, Young-Hoon Lee, Hye-Yeon Kim, Jung-Kil Lee, Min-Ho Shin, Shin-Seok Lee

**Affiliations:** 1 Division of Rheumatology, Department of Internal Medicine, Chonnam National University Medical School & Hospital, 42 Jebong-ro, Dong-gu, Gwangju, 61469 Republic of Korea; 2 Department of Biomedical Sciences, Chonnam National University Medical School, Gwangju, Republic of Korea; 3 Department of Preventive Medicine, Chonnam National University Medical School, 160 Baekseo-ro, Dong-gu, Gwangju, 61469 Republic of Korea; 4 Jeonnam Regional Cancer Center, Chonnam National University Hwasun Hospital, Hwasun, Republic of Korea; 5 Department of Preventive Medicine & Institute of Wonkwang Medical Science, Wonkwang University College of Medicine, Iksan, Republic of Korea; 6 Gwangju-Jeonnam Regional Cardiocerebrovascular Center, Chonnam National University Hospital, Gwangju, Republic of Korea; 7 Department of Neurosurgery, Chonnam National University Medical School and Hospital, Gwangju, Republic of Korea

**Keywords:** Rheumatology, Signs and symptoms

## Abstract

Several studies have evaluated the association between serum adiponectin levels and knee and hand osteoarthritis (OA); mixed results have been reported. We investigated the relationship between OA and serum adiponectin levels according to the radiographic features of knee and hand OA. A total of 2402 subjects was recruited from the Dong-gu Study. Baseline characteristics were collected via a questionnaire, and X-rays of knee and hand joints were scored using a semi-quantitative grading system. The relationship between serum adiponectin levels and radiographic severity was evaluated by linear and logistic regression analysis. Subjects in the higher serum adiponectin levels tertiles were older and had a lower body mass index (BMI) than those in the lower tertiles. Regarding knee joint scores, serum adiponectin levels was positively associated with the total (P < 0.001), osteophyte (P = 0.003), and joint space narrowing (JSN) scores (P < 0.001) after adjustment for age, sex, BMI, smoking, alcohol consumption, education, and physical activity. In terms of hand joint scores, no association was found between serum adiponectin levels and the total, osteophyte, JSN, subchondral cyst, sclerosis, erosion, or malalignment score after the above-mentioned adjustments. Similarly, subjects with serum adiponectin levels above the median had higher total radiographic scores in the knee joints, but not in the hand joints, after adjustment. An increased serum adiponectin levels was associated with a higher radiographic score in the knee joint, but not in the hand joint, suggesting the involvement of different pathophysiologic mechanisms in the development of OA between those joints.

## Introduction

Osteoarthritis (OA) is the most common form of arthritis, affecting the knees, hips, and hands in the appendicular joints^1^. OA is characterized by progressive cartilage degradation, bone remodeling, osteophyte formation, and synovial inflammation, which lead to pain, stiffness, swelling, and disability. Although the risk factors for the occurrence and progression of OA differ on the basis of the joints involved, obesity, age, sex, physical activity, previous injury, and genetic factors are implicated in its development and progression in all joints^2^. Obesity is the most important risk factor because it not only plays a mechanical role by increasing joint load, but also metabolic and inflammatory roles as a result of the secretion by adipose tissue of proinflammatory factors, such as adipokines, cytokines, and chemokines^3^.

Adipokines are soluble factors that predominantly originate from adipocytes and are associated with obesity-related inflammation. Adiponectin is secreted in large quantities by adipose tissue and plays important roles in the regulation of glucose and lipid metabolism^4^. In arthritis models and joint tissues, adiponectin plays roles in the initiation and progression of OA, and exerts pro- or anti-inflammatory effects depending on the tissue and cell types^5^. Although the circulating adiponectin level was reported be elevated in patients with OA compared to healthy controls in two recent meta-analyses^6,7^, the role of adiponectin in the pathogenesis of OA is controversial. Several studies have evaluated the association between plasma and synovial fluid adiponectin levels and the severity of OA in the knee and hand based on radiographic findings. Studies examining the correlation of the adiponectin level with the severity of knee joint OA based on the Kellgren-Lawrence grade have yielded inconsistent results (positive associations in two studies^8,9^, negative associations in two others^10,11^, and no association in three studies^12–14^. Similarly, two studies found an inverse association between the adiponectin level and radiographic hand OA^15,16^, but two others found no such association^17,18^. These varying results may be attributable to the small numbers of subjects and use of a low-accuracy radiographic grading method, such as the Kellgren-Lawrence grade. To overcome these limitations of previous studies, we took advantage of the availability of a large, population-based cohort to evaluate the relationship between the adiponectin level and radiographic severity of knee and hand OA using a novel, semi-quantitative grading system^19^. The aim of this study was to investigate the association of serum adiponectin levels with the radiographic features of knee and hand OA.

## Methods

### Population and study design

The subjects were from the Dong-gu Study, a population-based cohort study performed in South Korea. The study was conducted from 2007 to 2010 in the Dong-gu area of Gwangju Metropolitan City; 9260 subjects aged ≥ 50 years participated in the study^20^. To identify risk factors for OA, an ancillary study was conducted on 2516 subjects who participated in the Dong-gu Study in 2009. X-rays of the knees and hands of 2489 subjects were obtained. We included 2402 subjects in the final analysis after excluding 51 subjects with a history of total knee arthroplasty surgery or knee amputation, 15 with missing data on smoking and drinking status, and 21 with missing data on adiponectin levels. The study was approved by the Institutional Review Board of Chonnam National University Hospital (IRB approval no. CNUH-2019-335), and all patients provided written informed consent at the time of enrollment. This study was conducted in accordance with the Declaration of Helsinki and the Good Clinical Practice guidelines.

### Covariates

We collected information on the subjects’ baseline characteristics—including smoking, alcohol drinking, education and physical activity—by means of questionnaires. Body mass index (BMI) was calculated as the weight in kilograms divided by the height in meters squared. Smoking status was categorized as never smoker (< 100 lifetime cigarettes and not smoking currently), ex-smoker (> 100 lifetime cigarettes and not smoking currently), or current smoker (> 100 lifetime cigarettes and smoking now). Alcohol consumption in the past 12 months was used as the criterion to distinguish current alcohol drinkers from non-drinkers. Educational attainment was classified as high school or above high school. Physical activity was classified as regular or irregular according to the frequency of engagement in physical training/leisure activities during a typical week.

### Assay of serum adiponectin levels

Serum adiponectin levels were measured by enzyme-linked immunosorbent assay (AdipoGen, Incheon, South Korea) and divided into three tertiles: < 12.08 μg/mL, 12.09–19.69 μg/mL, and ≥ 19.72 μg/mL. The intra-assay coefficient was 5.2% and the inter-assay coefficient of variance was 7.2%.

### Radiographic assessment

Anteroposterior X-rays of the hands and knees were obtained with the subject in the standing position. The X-ray images were evaluated by two independent observers using a semi-quantitative grading system^21^. The intraobserver (kappa, 0.85–0.92) and interobserver (kappa, 0.79–0.89) reliability were both good.

We described the semi-quantitative grading system previously^22^. In brief, radiographic features were noted for the hand (distal interphalangeal joint [DIP], proximal interphalangeal joint [PIP], carpometacarpal joint [CMC], interphalangeal joint of the thumb [IP], and naviculotrapezial joint [NTJ]) and knee (medial compartment, lateral compartment, tibial component, and femoral component). The degree of damage was graded as 0 to 3 (0, normal; 1, mild; 2, moderate; 3, severe), or as absent (0) or present (1). For the hand, osteophyte and joint space narrowing (JSN) scores were obtained for the DIP (0–3), PIP (0–3), CMC (0–3), IP (0/1), and NTJ (0/1); sclerosis and malalignment scores were obtained for the DIP (0/1), PIP (0/1), and CMC (0/1); subchondral cyst scores were obtained for the PIP (0/1) and CMC (0/1); and erosion scores were obtained for the DIP (0/1), DIP—central erosion (0/1), DIP—pseudowidening (0/1), PIP (0/1), and CMC (0/1). For the knee, osteophyte scores were obtained for the medial femoral condyle (0–3), medial tibial plateau (0–3), lateral femoral condyle (0–3), and lateral tibial plateau (0–3); JSN scores were obtained for the medial compartment (0–3) and lateral compartment (0–3); a medial tibial attrition score was also obtained (0–3, as well as medial tibial sclerosis (0–3) and lateral femoral sclerosis scores (0–3). The total score (max = 70) of the hand joint was given by the sum of the scores on the six subscales: the osteophyte score (max = 22), JSN score (max = 22), subchondral cysts score (max = 4), sclerosis score (max = 6), erosion score (max = 10), and malalignment score (max = 6). The total score (max = 42) of the knee joint was given by the sum of the scores on the four subscales: osteophyte score (max = 24), JSN score (max = 12), tibial attrition score (max = 2), and sclerosis score (max = 4).

### Statistical analysis

The general characteristics of the subjects are presented as means and standard deviation (SD) for continuous variables and as number (%) for categorical variables. Continuous variables were compared by one-way analysis of variance, and categorical variables by chi-squared test. Linear regression analysis was performed to assess the relationship between serum adiponectin levels and radiographic scores of the knee and hand joints after adjusting for age, sex, BMI, smoking, alcohol consumption, education, and physical activity. P-values for paired comparisons among adiponectin tertiles were adjusted using the Bonferroni correction. Values are provided as adjusted means ± SD. In addition, dichotomous logistic regression analyses were conducted to determine whether values above the median adiponectin levels were associated with increased radiographic severity based on the median radiographic scores. A value of P < 0.05 was taken to indicate statistical significance. Statistical analysis was performed using Stata software (version 14.2; Stata Corporation, College Station, TX, USA).

## Results

### Baseline characteristics stratified by serum adiponectin levels

The baseline characteristics and radiographic features of the knee and hand joints according to serum adiponectin levels tertile are presented in Table 1. Among the 2402 subjects, the mean age was 64 years (SD, 8.2 years) and the mean BMI was 24.4 kg/m^2^ (SD, 2.9 kg/m^2^); 1042 subjects (43.4%) were male. Subjects in the higher serum adiponectin tertiles were older (mean age, 64.9 years) and had a lower BMI (mean, 23.8 kg/m^2^) than those in the lower tertiles. In addition, the proportions of subjects who were male (24.3%), current smokers (8.1%), current alcohol drinkers (40.1%), had an educational level of above high school (26.0%), and partook in regular physical activity (13.9%) were lower in subjects in the higher serum adiponectin tertiles than in those in the lower tertiles.

**Table 1:** Baseline subject characteristics according to serum adiponectin levels (μg/mL).

	Total	Tertile 1 (< 12.08)	Tertile 2 (12.09–19.69)	Tertile 3 (≥ 19.72)	P value
Number	2402	802	800	800	
Serum adiponectin levelss, μg/mL	17.3 ± 9.3	8.3 ± 2.6	15.7 ± 2.2	27.9 ± 7.3	< 0.001
Age, years	64 ± 8.2	62.8 ± 7.8	64.2 ± 8.0	64.9 ± 8.7	< 0.001
BMI, kg/m^2^	24.4 ± 2.9	24.8 ± 2.7	24.6 ± 2.8	23.8 ± 3.1	< 0.001
Sex (male) (%)	1042 (43.4)	513 (64.0)	335 (41.9)	194 (24.3)	< 0.001
Current smoker (%)	293 (12.2)	144 (18.0)	84 (10.5)	65 (8.1)	< 0.001
Current alcohol drinker (%)	1197 (49.8)	489 (70.0)	387 (48.4)	321 (40.1)	< 0.001
Education, above high school (%)	776 (32.3)	323 (40.3)	245 (30.6)	208 (26.0)	< 0.001
Physical activity (%)	394 (16.4)	167 (20.8)	116 (14.5)	111 (13.9)	< 0.001
**Knee**					
Total score	14.5 ± 6.9	13.2 ± 6.4	14.9 ± 7.0	15.3 ± 7.1	< 0.001
Osteophyte score	7.6 ± 3.7	7.0 ± 3.4	7.9 ± 3.8	7.9 ± 3.9	< 0.001
JSN score	6.0 ± 2.6	5.5 ± 2.5	6.2 ± 2.5	6.5 ± 2.5	< 0.001
Tibial attrition score	0.2 ± 0.6	0.2 ± 0.6	0.3 ± 0.6	0.2 ± 0.6	0.136
Sclerosis score	0.6 ± 0.9	0.5 ± 0.9	0.6 ± 0.9	0.7 ± 1.0	0.010
**Hand**					
Total score	16.9 ± 6.3	16.1 ± 5.8	17.0 ± 6.3	17.7 ± 6.6	< 0.001
Osteophyte score	6.5 ± 2.1	6.5 ± 2.2	6.5 ± 2.1	6.4 ± 2.0	0.448
JSN score	8.6 ± 3.3	8.0 ± 3.1	8.6 ± 3.3	9.1 ± 3.4	< 0.001
Subchondral cyst score	1.0 ± 1.3	0.8 ± 1.1	1.0 ± 1.2	1.3 ± 1.4	< 0.001
Sclerosis score	0.4 ± 0.9	0.4 ± 0.9	0.4 ± 0.9	0.4 ± 0.8	0.908
Erosion score	0.3 ± 1.0	0.3 ± 1.0	0.3 ± 0.9	0.3 ± 1.0	0.699
Malalignment score	0.1 ± 0.5	0.1 ± 0.5	0.1 ± 0.5	0.1 ± 0.5	0.942

Unless otherwise indicated, values are means ± standard deviation.

*BMI* body mass index; *JSN* joint space narrowing.

Regarding the radiographic scores of the knee joints, subjects in the higher serum adiponectin tertiles had a significantly higher total score (P < 0.001), osteophyte score (P < 0.001), JSN score (P < 0.001) and sclerosis score (P = 0.010) than those in the lower tertiles. However, the tibial attrition score did not differ among serum adiponectin tertiles (P = 0.136). Regarding the hand joint scores, subjects in the higher serum adiponectin tertiles had a significantly higher total score, JSN score, and subchondral cyst score than those in the lower tertiles (all P < 0.001). However, the osteophyte, sclerosis, erosion, and malalignment scores did not differ among serum adiponectin tertiles.

### Association between the tertiles of serum adiponectin levels and knee and hand OA

Table 2 shows the relationships between serum adiponectin levels and total and individual radiographic scores of the knee and hand joints. In multiple linear regression analyses adjusted for age, sex, BMI, smoking, alcohol consumption, education level, and physical activity, serum adiponectin levels were positively associated with the total (P < 0.001), osteophyte (P = 0.003), and JSN (P < 0.001) scores in knee joints. However, in multiple linear regression analyses, there was no significant difference in the score for any hand joint parameter among the serum adiponectin tertiles after adjusting for age, sex, BMI, smoking, alcohol consumption, education level, and physical activity.

**Table 2:** Associations of serum adiponectin levels (μg/mL) tertiles with total and individual radiographic scores of knee and hand osteoarthritis.

	Tertile 1 (< 12.08)	Tertile 2 (12.09–19.69)	Tertile 3 (≥ 19.72)	P value
**Knee joint**				
Total score	13.79 ± 0.22	14.72 ± 0.21	14.96 ± 0.22	< 0.001
Osteophyte score	7.28 ± 0.12	7.79 ± 0.12	7.83 ± 0.12	0.003
JSN score	5.70 ± 0.08	6.10 ± 0.08	6.30 ± 0.08	< 0.001
Tibial attrition score	0.23 ± 0.02	0.24 ± 0.02	0.22 ± 0.02	0.808
Sclerosis score	0.58 ± 0.03	0.60 ± 0.03	0.61 ± 0.03	0.879
**Hand joint**				
Total score	16.90 ± 0.19	16.85 ± 0.18	16.99 ± 0.19	0.854
Osteophyte score	6.40 ± 0.07	6.51 ± 0.07	6.54 ± 0.07	0.380
JSN score	8.58 ± 0.10	8.55 ± 0.10	8.63 ± 0.10	0.871
Subchondral cyst score	1.01 ± 0.04	0.98 ± 0.04	1.10 ± 0.04	0.096
Sclerosis score	0.39 ± 0.03	0.40 ± 0.03	0.39 ± 0.03	0.999
Erosion score	0.35 ± 0.03	0.28 ± 0.03	0.24 ± 0.03	0.109
Malalignment score	0.16 ± 0.02	0.13 ± 0.02	0.10 ± 0.02	0.066

Values are adjusted for age, sex, body mass index, smoking, alcohol consumption, educational level, and physical activity.

P values are for comparisons among all serum adiponectin levels tertiles.

*JSN* joint space narrowing.

### Associations of serum adiponectin levels above and below the median with knee and hand OA

Table 3 shows the relationships of serum adiponectin levels above and below the median with the total radiographic scores of the knee and hand joints based on the median radiographic scores. In multivariate logistic regression analysis adjusted for age, sex, BMI, smoking, alcohol consumption, education level, and physical activity, subjects with serum adiponectin levels above the median had higher total radiographic scores in the knee joints (P = 0.049), but not in the hand joints. Regarding the individual radiographic scores (Supplementary Table S1), adiponectin levels above the median were associated with the osteophyte (P = 0.018) and JSN (P = 0.022) scores in knee joints, and erosion (P = 0.026) and malalignment (P = 0.046) scores in hand joints, after adjustment.

**Table 3:** Multivariate logistic regression analysis of high total score of knee and hand osteoarthritis based on the median radiographic scores.

Number (%)	Odds ratio (95% confidence interval)	P value
**Knee joint**		
Adiponectin (low vs high)*	1.21 (1.00–1.46)	0.049
Age (1 year)	1.11 (1.10–1.13)	< 0.001
Body mass index (1 kg/m^2^)	1.11 (1.08–1.15)	< 0.001
Sex (male vs female)	1.17 (0.94–1.46)	0.164
Current smoker (yes vs no)	1.20 (0.90–1.61)	0.218
Current alcohol drinker (yes vs no)	0.92 (0.77–1.12)	0.417
Education, above high school (yes vs no)	0.64 (0.52–0.78)	< 0.001
Physical activity (yes vs no)	0.87 (0.68–1.10)	0.243
**Hand joint**		
Adiponectin (low vs high)*	1.00 (0.82–1.23)	0.973
Age (1 year)	1.16 (1.15–1.18)	< 0.001
Body mass index (1 kg/m^2^)	1.04 (1.01–1.08)	0.012
Sex (male vs female)	1.99 (1.56–2.54)	< 0.001
Current smoker (yes vs no)	1.15 (0.84–1.58)	0.387
Current alcohol drinker (yes vs no)	1.02 (0.83–1.24)	0.884
Education, above high school (yes vs no)	0.78 (0.63–0.97)	0.024
Physical activity (yes vs no)	0.70 (0.54–0.91)	0.008

*Adiponectin levels were dichotomized by the median values.

## Discussion

In this cross-sectional study, serum adiponectin levels were positively associated with the radiographic severity of OA in the knee joints, but not in the hand joints. Among the knee OA parameters, the adiponectin level was associated with the osteophyte, JSN, and total scores. To our knowledge, this is the largest study to investigate the relationship between serum adiponectin levels and radiographic severity of OA using a semi-quantitative grading system.

In this study, serum adiponectin levels were positively associated with the radiographic severity of OA of the knee joints, and particularly with the osteophyte and JSN scores. Prior studies of the association between the blood adiponectin level and radiographic severity of knee OA produced conflicting results. In a Finish study of 35 male patients with knee OA, the plasma adiponectin level was significantly higher in patients with radiographically severe OA than in those with mild OA^8^. In a Turkish study of 60 patients with knee OA, serum adiponectin levels were positively correlated with the Kellgren-Lawrence grade^9^. However, in a Thai study^10^ and the Anhui OA study^11^, the circulating adiponectin level was negatively associated with disease severity, as measured by the Kellgren-Lawrence grade, suggesting a protective role for adiponectin against knee OA. Interestingly, no association was found between the blood adiponectin level and severity of knee OA in the Framingham Offspring cohort study^14^, nor in Greek^12^, and Dutch studies^13^. These conflicting results may be attributable to small numbers of OA patients, use of radiographic grading systems with low accuracy (such as the Kellgren-Lawrence grade), inadequate control for confounding variables in the statistical analysis, and the use of different methods to measure isoforms of adiponectin. In comparison with previous studies, we recruited a large number (2402) of subjects from the Dong-gu study, obtained detailed information on the radiographic features of OA using a semi-quantitative grading system, and adjusted for relevant confounding variables in the statistical analysis. Although our finding of a positive association of adiponectin with the radiographic severity of knee OA requires confirmation in a prospective study, it provides insight into the role of adiponectin in the initiation and progression of knee OA.

Serum adiponectin levels were not associated with the radiographic severity of OA of the hand joints. A longitudinal study of 164 patients with hand OA reported that a higher level of adiponectin was associated with a lower risk of radiographic progression^15^. Another longitudinal study of 224 patients with hand OA investigated the associations between total and high-molecular-weight (HMW) adiponectin levels with the radiographic severity of hand OA^16^. The total adiponectin level was inversely associated with radiographic progression, but the HMW adiponectin level was not associated with progression. In contrast, in two cross-sectional studies^17,18^, serum adiponectin levels were not associated with the radiographic severity of hand OA. Our findings are in accordance with the results of cross-sectional studies, but not with those of longitudinal studies; this suggests that the differences are attributable to period and cohort effects^23^. Further studies are needed to determine if there is a causal relationship between serum adiponectin levels and radiographic severity of OA of the hand.

In this study, serum adiponectin levels were associated with the radiographic severity of OA in the knee joints, but not in the hand joints. Two hypotheses can be formulated that may explain the difference. First, obesity has a greater effect on knee OA than on hand OA^24^. In our previous study of the Dong-gu cohort, body weight was significantly associated with the radiographic severity of knee, but not hand, OA^25^. Because adiponectin originates predominantly from adipose tissue, it can be postulated that weight-bearing knee joints are more closely associated with the adiponectin level than are non-weight-bearing hand joints. Second, the expression level of adiponectin receptors differs among the joints. Adiponectin acts via two receptors: AdipoR1, predominantly found in skeletal muscle; and AdipoR2, mainly present in the liver^26^. The skeletal muscle mass of knee joints is greater than that of hand joints, so the role of adiponectin may differ among joints.

The strengths of our study included its inclusion of a large population-based cohort, detailed radiographic grading, and simultaneous evaluation of knee and hand joints. However, the study also had several limitations. First, the design was cross-sectional rather than longitudinal; therefore, the results are correlational and causality cannot be determined. Second, although many potential confounding factors were adjusted for, residual confounding effects of unmeasured variables may have introduced bias. Third, the adiponectin level was measured systemically, so may not reflect the intraarticular adiponectin level.

## Conclusion

In this population-based study, serum adiponectin levels were positively associated with the radiographic severity of OA in knee joints, but not in hand joints. Our findings indicate that different pathophysiologic mechanisms underlie the initiation and progression of OA of the knee and hand joints.

## Supplementary Information

Supplementary Information.

## Data Availability

Full original protocol and dataset can be accessed upon request for academic researchers by contacting Professor Shin-Seok Lee (shinseok@chonnam.ac.kr).
