# Supplementary material for: Increased adiponectin levels are associated with higher radiographic scores in the knee joint, but not in the hand joint
Source: Sci Rep. 2021 Jan 19;11:1842. doi: 10.1038/s41598-021-81513-z (PMC7815782; doi:10.1038/s41598-021-81513-z)
Supplement: Supplementary file 1 — Supplementary Information. [file 41598_2021_81513_MOESM1_ESM.docx]

Supplementary Table 1. Logistic regression analyses to determine whether values above the median adiponectin levels were associated with increased radiographic severity based on the median radiographic scores.

| Number (%) | Low adiponectin  (below median, n=1201) | High adiponectin  (above median, n=1201) | Odds ratio (95% confidence interval) | P value |
| --- | --- | --- | --- | --- |
| Knee joint |  |  |  |  |
| Total score | 521 (43.4) | 613 (51.0) | 1.21 (1.00-1.46) | 0.049 |
| Osteophyte score | 452 (37.6) | 540 (45.0) | 1.25 (1.04-1.51) | 0.018 |
| JSN score | 405 (33.7) | 526 (43.8) | 1.26 (1.03-1.53) | 0.022 |
| Tibial attrition score | 178 (14.8) | 192 (16.0) | 0.89 (0.69-1.14) | 0.353 |
| Sclerosis score | 415 (34.6) | 440 (36.6) | 0.92(0.76-1.11) | 0.385 |
| Hand joint |  |  |  |  |
| Total score | 458 (38.1) | 552 (46.0) | 1.00 (0.82-1.23) | 0.973 |
| Osteophyte score | 544 (45.3) | 533 (44.4) | 1.11 (0.92-1.33) | 0.274 |
| JSN score | 477 (39.7) | 558 (46.5) | 0.87 (0.71-1.05) | 0.146 |
| Subchondral cyst score | 313 (26.1) | 474 (39.5) | 1.12 (0.91-1.38) | 0.289 |
| Sclerosis score | 251 (20.9) | 256 (21.3) | 1.02 (0.82-1.26) | 0.893 |
| Erosion score | 145 (12.1) | 154 (12.8) | 0.73 (0.55-0.96) | 0.026 |
| Malalignment score | 100 (8.3) | 101 (8.4) | 0.72 (0.52-0.99) | 0.046 |

Values in the second and third columns are the number and percentage of subjects with high radiologic osteoarthritis scores in the low adiponectin and high adiponectin groups.

Odds ratios were adjusted for age, sex, body mass index, smoking, alcohol consumption, educational level, and physical activity.

Abbreviation: JSN, joint space narrowing.
